# Supplementary material for: Effectiveness and Safety of Ayurvedic Medicines in Type 2 Diabetes Mellitus Management: A Systematic Review and Meta-Analysis
Source: Front Pharmacol. 2022 Jun 8;13:821810. doi: 10.3389/fphar.2022.821810 (PMC9213670; doi:10.3389/fphar.2022.821810)
Supplement: Supplementary file 1 [file DataSheet1.zip › Supplementary Material/Forest and Funnel Plots/Syzygium cumini/FBG.pdf]

Syzygium cumini - FBG

Study	Intervention N	Intervention Mean	Intervention SD	Comparator N	Comparator Mean	Comparator SD	Mean Diff. with 95% CI	Weight (%)
Sahana 2010	15	140.70	26.60	10	127.50	11.50	13.20 [-4.37, 30.77]	47.05
Sidana 2016, Sidana 2017	50	121.00	15.14	49	143.00	11.24	-22.00 [-27.26, -16.74]	52.95
**Overall**							-5.44 [-39.87, 29.00]	

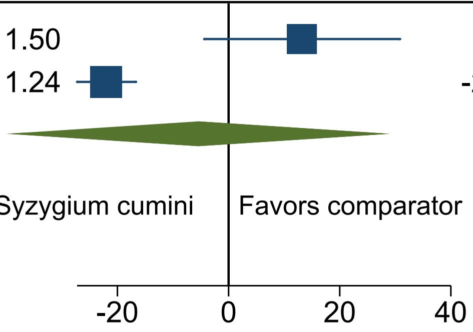

Heterogeneity: $\tau^2 = 575.71$, $I^2 = 92.93\%$, $H^2 = 14.14$

Test of $\theta_i = \theta_j$: Q(1) = 14.14, p = 0.00

Test of $\theta = 0$: z = -0.31, p = 0.76

Random-effects REML model
